# Endoscopic Vacuum-Assisted Closure Therapy in Patients with Anastomotic Leakage after Esophagectomy: A Single-Center Experience

**DOI:** 10.1155/2018/1697968

**Published:** 2018-04-04

**Authors:** Soo Min Noh, Ji Yong Ahn, Jeong Hoon Lee, Hwoon-Yong Jung, Zeead AlGhamdi, Hyeong Ryul Kim, Yong-Hee Kim

**Affiliations:** ^1^Department of Internal Medicine, Asan Digestive Disease Research Institute, Asan Medical Center, University of Ulsan College of Medicine, Seoul, Republic of Korea; ^2^Department of Gastroenterology, Asan Digestive Disease Research Institute, Asan Medical Center, University of Ulsan College of Medicine, Seoul, Republic of Korea; ^3^Department of Thoracic and Cardiovascular Surgery, Asan Medical Center, University of Ulsan College of Medicine, Seoul, Republic of Korea

## Abstract

**Aim:**

To study the efficacy of E-VAC therapy for patients with anastomotic leakage after esophagectomy.

**Methods:**

Between January 2013 and April 2017, 12 patients underwent E-VAC therapy for the management of postoperative leakage. Their clinical features and endoscopic procedure details, therapy results, adverse events, and survival were investigated.

**Results:**

All 12 patients were male and the median age was 57 years (interquartile range 51.5–62.8 years). The reasons for esophageal surgery were esophageal cancer (83.3%), gastrointestinal stromal tumor (8.3%), and esophageal diverticulum (8.3%). Prior to E-VAC therapy, 6 patients had undergone failed primary surgical repair and the median duration from esophagectomy to leakage discovery was 13.5 days (IQR 6–207 days). The median duration of E-VAC therapy was 25 days (IQR 13.5–34.8 days) and the average sponge exchange rate was 2.7 times during the treatment period. After E-VAC therapy, 8 patients (66.7%) had complete leakage closure, 3 (25%) had a decreased leakage size, and 1 (8.3%) was unchanged. The three patients with a decreased leakage size after E-VAC therapy were treated with endoscopic and conservative management without further surgery.

**Conclusion:**

With proper patient selection, E-VAC therapy is a feasible and safe method for the treatment of anastomotic leakage after esophagectomy.

## 1. Introduction

Anastomotic leakages occur at a rate of about 8% to 13% after esophageal surgery [1–4]. Because postoperative esophageal leakages are life-threatening events, the purpose of treatment is to prevent the leakage from affecting the perforation area and thereby protect gastrointestinal tract functions and ensure proper nutrition. Although surgical management is considered the primary treatment, it is often difficult to perform given the potential morbidity risks associated with reoperation. As alternatives, many patients have been treated with various endoscopic techniques [5–7]. However, no uniform endoscopic method exists for correcting postoperative anastomotic leakage. Among the many endoscopic methods tried, endoscopic vacuum-assisted closure (E-VAC) therapy is reported to be a good method for the treatment of postoperative esophageal leakage [8–14]. However, patient and method characteristics varied slightly among the clinical centers and sufficient data from a large number of patients have not been reported because these cases are rare in most hospitals.

We therefore analyzed in this study the efficacy and safety of E-VAC therapy in patients with anastomotic leakage after esophageal surgery performed in a single tertiary center.

## 2. Patients and Methods

### 2.1. Patients

**Figure 1:**
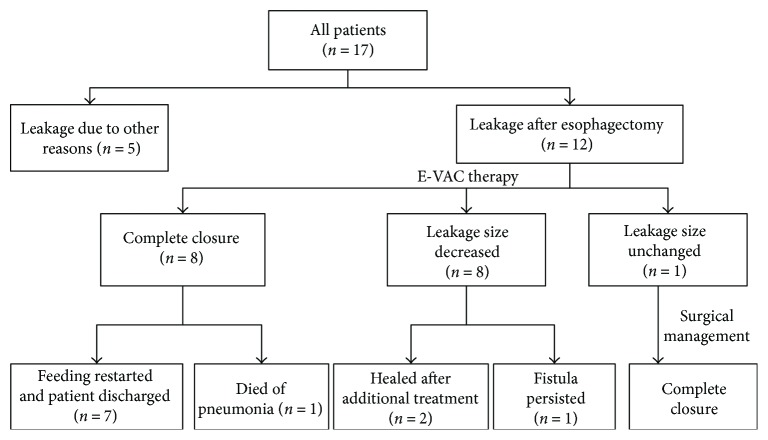
Flow chart of case enrollment.

Between January 2013 and April 2017, 17 patients underwent E-VAC therapy at Asan Medical Center, Seoul, Korea. Among the 17 patients, 12 patients underwent E-VAC therapy after esophagectomy. The other 5 patients who underwent E-VAC therapy for reasons other than post esophagectomy leakage were excluded (Figure 1). We retrospectively studied these patients and reviewed their clinicopathological features, radiologic studies, endoscopy reports, and clinical course. Outcome data included clinical success, evidence of need for an additional endoscopic procedure with or without surgery, and procedure-related complications. This study was approved by the Institutional Review Board of Asan Medical Center (IRB number: 2017-0736).

### 2.2. Diagnostic Method and Definitions

In the present study, radiological examinations to identify anastomotic leakage were routinely performed before food intake resumed. Leakage diagnosis was based on clinical presentation and radiological findings. Clinical diagnosis of anastomotic leakage was defined as physical findings suggesting pneumonia or as changes in content within the chest drain. Radiologic findings indicative of a leakage were defined as extraluminal extravasation of contrast on postoperative fluoroscopy or the presence of an infiltration around the anastomosis site and/or fistula tract formation on computed tomography (CT). Clinical success was defined as leakage closure confirmed by radiographic or endoscopic evaluation.

### 2.3. Leakage Management

There are no standard guidelines for the treatment of leakages after esophagectomy. The treatment methods varied among the patients in the current study according to the leakage size, timing of the diagnosis, and patient comorbidities. The attending surgeon decided whether to perform surgery or another medical treatment, including endoscopic procedures. In some cases, the surgery was performed first if primary repair was feasible. E-VAC therapy was then performed after consultation with an expert endoscopist when it was judged that the esophagoduodenoscopic assessment indicated the need for E-VAC therapy. During endoscopy, the distance from the incisors, defect diameter, and cavity shape were evaluated. All endoscopic interventions were performed by two expert endoscopists (JHL and JYA). During the E-VAC, all patients were treated with conservative treatment, which included nil by mouth, systemic antibiotics, total parental nutrition, and/or adequate external drainage.

### 2.4. The E-VAC Therapy Method

**Figure 2:**
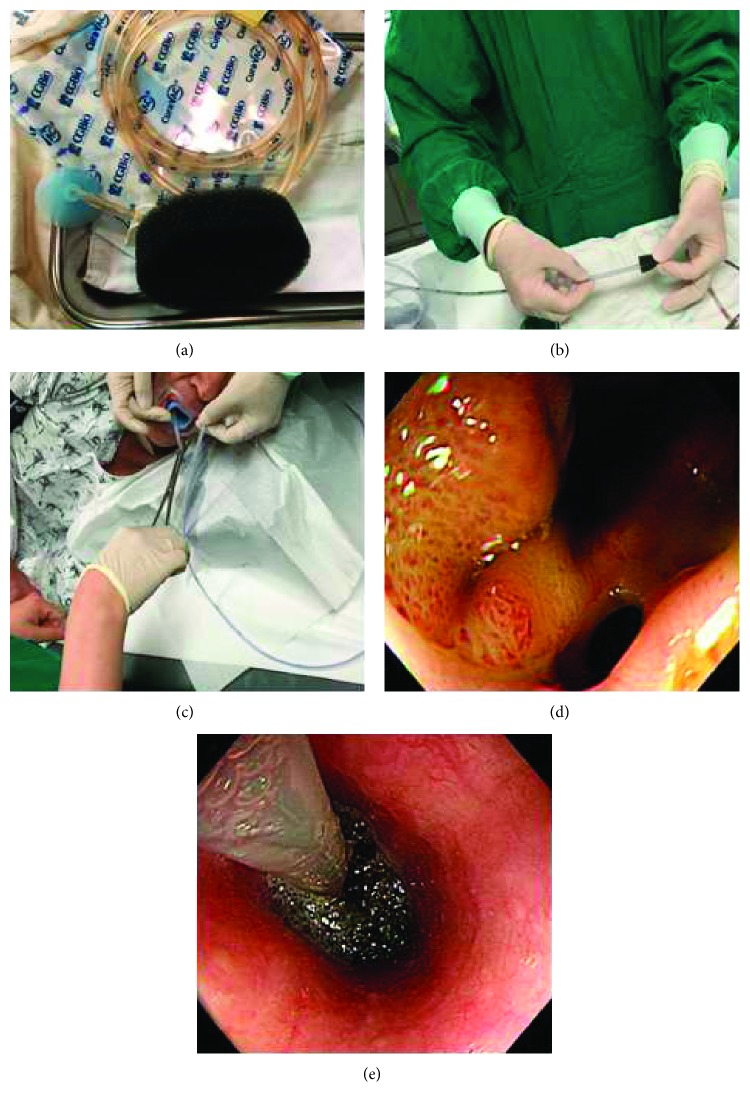
Steps in the endoscopic vacuum-assisted closure procedure. (a) The vacuum-assisted closure kit (CuraVAC; CGBio, Hwaseong, Korea) comprises a polyurethane sponge, adhesive drapes, and connector tubing. (b) A nasogastric tube with a polyurethane sponge head. The form and size of the sponge is similar to those of the anastomotic leakage. (c) A pulled-out nasogastric tube is connected to a polyurethane sponge. (d) Anastomotic leakage was noted on endoscopy (GIF-H290; Olympus, Tokyo, Japan). (e) The nasogastric tube embedded with a polyurethane sponge was placed in the anastomotic leakage area.

We used a single-channel gastroscope (GIF-H260 or GIF-H290; Olympus, Tokyo, Japan), nasogastric tube, and standard VAC kit (CuraVAC®; CGBio Inc., Hwaseong, Korea). The VAC kit comprises a medical-grade polyurethane sponge, adhesive drapes for sealing, and connector tubing inside a sterile pouch (Figure 2(a)). The procedure was performed under conscious sedation with intravenous administration of midazolam. We prepared a nasogastric tube and an appropriately sized piece of polyurethane sponge (Figure 2(b)). The size of the sponge is made to fit the size and the location of the cavity. Due to the size limitation of passing through the upper esophageal sphincter, most of them were made in sizes ranging from 2.0 × 2.0 cm to 3.0 × 3.0 cm. A nasogastric tube was inserted and retrieved through the mouth to attach the sponge (Figure 2(c)). A piece of sponge is cut to the appropriate size and positioned at the tip of the L-tube. Then, the sponge is sutured onto the L-tube tip using silk to connect and secure it. After the leakage site was identified (Figure 2(d)), the nasogastric tube with the polyurethane sponge was inserted into the esophagus and an endoscopic examination was performed to help in the correct placement of the tube and sponge with the use of a pair of forceps (Figure 2(e)). Usually, the intracavitary insertion of the drainage tube has been preferred if the sponge can fill the cavity properly. However, if the placement of the sponge in the cavity was difficult due to the small size or difficult location, it was placed in the esophageal lumen adjacent to the defect. After inserting the sponge into the leak site, a suction of 80–125 mmHg was applied to remove the fluid which prevents reepithelization. If the suction works successfully, negative pressure is applied and it helps if the sponge can be fixed to the cavity to some extent. Then, the final nasogastric tube position on the nose is marked and care is taken so that it is not moved.

**Figure 3:**
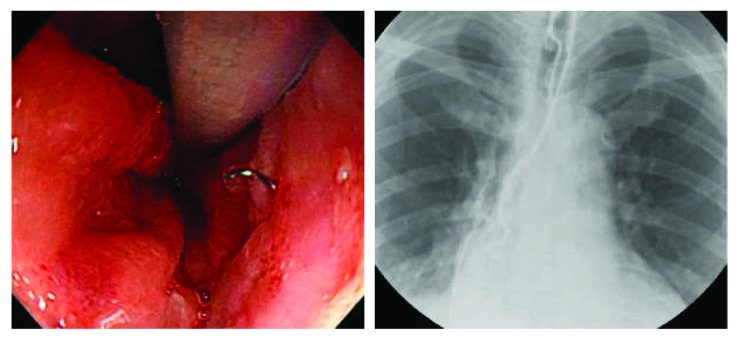
Endoscopic image and esophagography showing complete closure of the leakage after completion of the E-VAC therapy.

The change in the cavity was monitored and the sponge was endoscopically exchanged every 1 or 2 weeks if needed. If the leak appeared to be sealed on endoscopic examination, we removed the sponge and fluoroscopy was conducted to confirm complete closure of the leakage (Figure 3). If confirmed, the patient was allowed to sip water, before gradually advancing to a soft or regular diet. However, if the leakage persisted despite E-VAC therapy, clinicians discussed the case and decided whether to add treatment such as injection of fibrin glue (Beriplast; Aventis Behring Ltd., Marburg, Germany) or endoscopic clipping (Long Clip; Olympus Optical Co., Ltd.) or to continue with conservative management.

## 3. Results

### 3.1. Clinical Characteristics of the Study Patients

All 12 study patients were men, with a median age of 57 years (interquartile range (IQR) 51.5–62.8 years). The reasons for esophageal surgery were esophageal cancer (83.3%), gastrointestinal stromal tumor (GIST) (8.3%), and esophageal diverticulum (8.3%). Seven patients (58.3%) underwent open Ivor Lewis esophagectomy, four (33.3%) underwent robotic-assisted Ivor Lewis esophagectomy, and one (8.3%) underwent robotic esophageal diverticulectomy (Table 1).

### 3.2. Anastomotic Leakage Diagnosis

The median duration from esophagectomy to leakage discovery was 11 days (IQR 6.3–205.3 days). Nine patients (75%) reported symptoms at the time of diagnosis, and the other three patients were diagnosed by routine fluoroscopy. Leakages were diagnosed with fluoroscopy in 8 of the 12 patients, chest CT was used in three patients, and one patient who complained of dyspnea with increased contents within the chest tube was diagnosed without imaging. Three of the patients had previously been treated for anastomotic leakage immediately after the esophagectomy but the closed leakage opened again (Table 2).

### 3.3. Clinical Success of the E-VAC Therapy

The E-VAC therapy was initiated at a median of 11 days (IQR 5–54 days) after leakage diagnosis. Six patients (50%) had undergone failed primary surgical repair prior to E-VAC therapy, with persistence of the anastomotic leakage. In eight patients (66.7%), leakages were completely closed after E-VAC therapy with a median of five endoscopic interventions (IQR 2–8; four sponge insertions plus final sponge removal) and the median duration from the start of E-VAC therapy to final sponge removal was 28.5 days (IQR 15.0–34.8 days).

In three of the four remaining patients, the leakage size decreased after E-VAC therapy. In two of these patients, the leakage improved with further endoscopic management and conservative treatment. One patient had been treated with additional endoscopic clipping, but the fistula persisted. The remaining patient who had no response to E-VAC therapy was healed through surgical treatment. Details are given in Table 3 and Figure 1.

### 3.4. E-VAC Therapy-Related Complications and Death

The median follow-up duration was 12.9 months (IQR 1.2–18.6 months). There were no serious procedure-related complications and two patients had procedure-related complications that could be managed by additional medical treatment. In one patient, a symptomatic esophageal stricture was diagnosed and successfully treated with fluoroscopically guided balloon dilatation after 5 months of E-VAC therapy. In another patient, E-VAC therapy was stopped after 21 days due to bleeding at the anastomosis site. The bleeding was successfully treated with sponge removal and follow-up fluoroscopy revealed no more leakage of contrast media at the esophagocolonic anastomotic site. Although the leakage was closed, the patient died of persistent aspiration pneumonia 2 weeks later.

## 4. Discussion

In our present series of 12 patients who underwent E-VAC therapy for anastomotic leakage after esophagectomy, the clinical success rate was 66.7% (8/12) and 91.7% (11/12) of these cases showed a decreased leakage size, avoiding the need for additional surgery. The E-VAC therapy failed in one patient (8.3%) whose leakage was closed after surgical management. General anesthesia was not required and all procedures were performed safely using intravenous administration of midazolam. No major procedure-related complications were detected; the two patients who had an esophageal stricture and bleeding at anastomotic sites improved after proper management. These results suggest that, with careful case selection, E-VAC therapy can be an appropriate therapeutic option for the management of postoperative leakage that avoids further surgical intervention.

As an alternative treatment for anastomotic leakage after esophagectomy, E-VAC therapy was first introduced in 2008 by Wedemeyer et al. [15]. Compared with other endoscopic treatments, E-VAC therapy promotes healing of the anastomotic leakage by increasing vascular perfusion and enhancing the formation of granulation tissue [16]. According to previous data on 41 patients with postoperative leakage from the esophagus [8–11, 13, 15, 17], E-VAC therapy showed clinical success rates of between 66.7% and 100% (overall 90.2% (37/41)). The results of other studies are summarized in Table 4.

We found that the clinical success rate was considerably lower than that of other previous reports. In our study, six patients had already undergone failed primary closure before E-VAC therapy and other endoscopic management options were either impossible or deemed unfit. Thus, except for one patient, we chose E-VAC therapy as a treatment until it was changed because it was judged ineffective. Therefore, it is likely that we were offering E-VAC therapy to less well patients with more complex problems than in other series. Under difficult conditions, in 11 patients, additional surgical management could be avoided through E-VAC therapy and conservative treatment. Because surgical intervention for postoperative leakage is associated with a high risk of mortality and morbidity, E-VAC therapy was feasible in almost all patients.

There were no differences in leakage detection time after esophagectomy (176.0 ± 370.1 days versus 105.5 ± 187.8 days), duration from time of leakage diagnosis to E-VAC therapy (39.4 ± 60.6 days versus 42.5 ± 73.7 days), and duration of E-VAC therapy (25.8 ± 12.3 days versus 27.5 ± 23.6 days) between the successful and unsuccessful groups. According to other studies, failure is more likely in the case of chronic, larger, and/or loculated cavities [13]. Hence, these earlier reports recommended the evaluation of the cavity characteristics and determination of whether E-VAC therapy was indicated. Although, in our present study, there were no differences in patient characteristics between those who underwent successful or unsuccessful E-VAC therapy, we think that E-VAC therapy is less effective in a large leakage which cannot be covered by a swallow-to-mouth-sized sponge, in an opening which has lots of secretion such as the esophagobronchial fistula, and in a location where peristalsis exists. Therefore, the best-suited indication of E-VAC therapy is a less than 4 cm sized sealed-off leak lesion which is located on a relatively fixed site such as an anastomosis site. Further analysis with a larger number of patients in a multicenter setting should be performed to more precisely determine the clinical outcomes and indications for E-VAC therapy in patients with leakage after esophagectomy.

One of the limitations of E-VAC therapy is the need for repeated endoscopic procedures. Our current case studies required fewer sponge changes than reported for other patient series but there was no difference in the treatment period. Therefore, we recommend endoscopic sponge exchange at proper intervals according to patient comfort.

Our current study had limitations associated with its small number of subjects and retrospective design. In addition, our comparison between the successful and unsuccessful groups was limited. Nevertheless, we expect that our findings will provide an impetus for a future meta-analysis of the use of endoscopic therapy in the management of postoperative anastomotic leakage.

In conclusion, E-VAC therapy is a technically feasible and safe treatment option in patients with anastomotic leakage after esophagectomy. With proper implementation of additional supportive treatments, E-VAC therapy will be able to replace surgical management in carefully selected patients. Future studies involving a greater number of patients are needed to evaluate the efficacy of E-VAC therapy.

## Figures and Tables

**Table 1:** Baseline characteristics of the study patients (*n* = 12).

Variable	No. of patients
Male	12 (100%)
Median age (years)	57.0 (51.5–62.75)
Etiology	
Esophageal cancer	10 (83.3%)
GIST	1 (8.3%)
Esophageal diverticulum	1 (8.3%)
Methods of primary surgery	
Open Ivor Lewis esophagectomy	7 (58.3%)
Robotic-assisted Ivor Lewis esophagectomy	4 (33.3%)
Robotic esophageal diverticulectomy	1 (8.3%)

Data are presented as a median value (interquartile range) or number (%). GIST, gastrointestinal stromal tumor.

**Table 2:** Anastomotic leakage diagnosis.

Variable	No. of patients
Median duration from esophagectomy to leakage (days)	11 (6.3–205.3)
Presence of symptoms	9 (75%)
Diagnostic modality	
Fluoroscopy	8 (66.7%)
Chest CT	3 (25%)
Other^a^	1 (8.3%)

Data are presented as a median value (interquartile range) or number (%). CT, computed tomography. ^a^By high clinical suspicion.

**Table 3:** Characteristics of the 12 study patients who underwent E-VAC therapy.

Patient	Etiology	Premanagement prior to E-VAC therapy	Opening size (cm)	Placement of E-VAC	E-VAC therapy duration (days)	Sponge exchanges *(n)*	E-VAC therapy result	Clinical outcome	Complication
1	Esophageal cancer	Failed primary closure	2	Intracavitary	27	1	Complete closure	Discharged	Anastomotic site stricture
2	Esophageal cancer	None	1	Intraluminal	43	6	Complete closure	Discharged	None
3	Esophageal cancer	Failed primary closure	0.5	Intraluminal	31	3	Complete closure	Discharged	None
4	Esophageal cancer	None	1	Intraluminal	5	0	Complete closure	Discharged	None
5	GIST	None	1	Intraluminal	13	1	Complete closure	Discharged	None
6	Esophageal cancer	Succeeded in primary closure but leakage opened again	1	Intraluminal	36	6	Complete closure	Discharged	None
7	Esophageal diverticulum	None	1.5	Intraluminal	30	4	Complete closure	Discharged	None
8	Esophageal cancer	Failed primary closure	0.5	Intraluminal	21	3	Complete closure	Died of pneumonia	Anastomotic site bleeding
9	Esophageal cancer	Failed repeated primary closures	1, 1^∗^	Intraluminal	62	5	Size decreased	Healed after further supportive tx.	None
10	Esophageal cancer	None	1.5	Intraluminal	15	1	Size decreased	Healed after supportive tx. & fibrin glue injection	None
11	Esophageal cancer	None	2.5	Intracavitary	23	2	Size decreased	Fistula persisted despite additional endoscopic clipping	None
12	Esophageal cancer	Failed primary closure	2.5, 1.5^∗^	Intracavitary	10	0	No change	Healed after surgical management	None

GIST, gastrointestinal stromal tumor; tx., treatment. ^∗^Two fistulas.

**Table 4:** E-VAC therapy clinical success rates reported in previous case series.

Study	No. of treated patients	Complete closure rate (%)
Kuehn et al. [8] (2012)	3	2/3 (67)
Weidenhagen et al. [9] (2010)	6	5/6 (83)
Ahrens et al. [10] (2010)	5	5/5 (100)
Bludau et al. [11] (2014)	5	5/5 (100)
Ooi et al. [13] (2016)	2	2/2 (100)
Wedemeyer et al. [15] (2008)	8	7/8 (88)
Schorsch et al. [17] (2013)	12	11/12 (92)
